# Equity and prediction of health resource allocation of traditional Chinese medicine in China

**DOI:** 10.1371/journal.pone.0290440

**Published:** 2023-08-24

**Authors:** Minghua Zhou

**Affiliations:** Department of administration office, Luzhou People’s Hospital, Luzhou, Sichuan, China; Universidad Nacional Autonoma de Nicaragua Leon, NICARAGUA

## Abstract

**Objective:**

To analyze the equity of health resource allocation of Traditional Chinese Medicine(TCM) and predict its development during the 14th Five-Year Plan period, and to provide a scientific basis for promoting the improvement of TCM service capacity.

**Methods:**

The Chinese Mainland (excluding Hong Kong, Macao and Taiwan) was divided into the Northeast, Eastern, Central and Western regions, and the number of TCM medical institutions, the number of TCM beds, practitioners (assistants) of TCM and Chinese pharmacists from 2016 to 2020 were selected as evaluation indicators, and the equity of health resource allocation of TCM was evaluated by Concentration index(CI), Theil index(T) and Health resource agglomeration degree (HRAD), and the development of health resource of TCM during the 14th Five-Year Plan period was predicted by grey prediction model GM (1,1).

**Results:**

The Concentration index of the number of TCM medical institutions and TCM beds is negative, and the allocation tends to the regions with low economic development level. The Concentration index of practitioners (assistants) of TCM and Chinese pharmacists is positive, and the allocation tends to the regions with higher economic development level. The number of TCM medical institutions, TCM beds, practitioners (assistants) of TCM and Chinese pharmacists’ Theil index allocated by geography is larger than that allocated by population, which indicates that the equity of TCM health resources allocated by population is better than that allocated by geography. The number of TCM medical institutions, practitioners (assistants) of TCM and Chinese pharmacists in between regions by population contributed more than 72% to the Theil index, indicating that the inequity mainly comes from between regions. The number of TCM medical institutions, TCM beds, practitioners (assistants) of TCM and Chinese pharmacists in within regions by geography contributed more than 80% to the Theil index, indicating that the inequity mainly comes from within regions. The HRAD in the Eastern and Central regions is greater than 1, indicating that the equity is better by geography. The HRAD in the Western region is less than 1, indicating insufficient equity by geography. The HRAD/PAD of the Central region (except for the number of TCM beds in 2020) is less than 1, indicating that it cannot meet the medical needs of the agglomerated population. The HRAD/PAD of the Western region (excluding for the Chinese pharmacists) is greater than 1, indicating that the equity is better than that of the agglomeration population.

**Conclusion:**

The number of TCM medical institutions and TCM beds tends to regions with low economic development levels, while the number of practitioners (assistants) of TCM and Chinese pharmacists tends to regions with high economic development levels. The equity of the allocation of TCM health resources by population is better than that by geography, and the inequity of the allocation by geography mainly comes from within region. The allocation of health resources of TCM in the four regions is different, and there is a contradiction between equity and actual medical needs.

## Introduction

Traditional Chinese Medicine (TCM) is a unique health resource in China and plays an important role in social and economic development [1]. To inherit, develop and make good use of TCM, it is conducive to give full play to the role of TCM in the all-round and full-cycle protection of people’s health.

In order to promote the healthy development of TCM, the Chinese government has always attached great importance to the development of TCM. The Notice of the State Council on the Printing and Distributing the Strategic Planning Outline for the Development of TCM (2016–2030) (GF[2016] No. 15) pointed out that by 2030 [2], the modernization level of the TCM management system and management capacity will be significantly improved, TCM services will be fully covered, and the capacity of TCM health service will be significantly enhanced. The Notice of the General Office of the State Council on the Printing and Distribution of the Fourteenth Five-Year Plan for the Development of TCM (GBF [2022] No. 5) pointed out that by 2025 [3], the health service capacity of TCM will be significantly enhanced, the high-quality development policy and system of TCM will be further improved, the revitalization and development of TCM will achieve positive results. In the context of attaching great importance to the inheritance and innovative development of TCM, it is important to analyze the allocation of TCM health resources and development projections in China.

Providing fair and accessible TCM health services in the whole life cycle is an important goal to meet the people’s enjoyment of TCM services. It is also an important part of ensuring health equity and achieving health for all [4]. To achieve the goals of the fourteenth five-year plan for the development of TCM, the premise is the rational allocation and effective use of health resources of TCM. With the rapid development of China’s social economy, the total amount of TCM health resources is insufficient, the urban-rural distribution is unreasonable, the regional allocation is unfair, and the relationship between medical and health system construction and medical demand is wrong [5]. As a result, TCM health services cannot meet the growing medical needs of the people. In particular, the sustainable development of the differences between regions in the allocation of TCM health resources has caused the imbalance between medical and health service regions [6], which has become a common feature of China’s and even the global health system [7]. Therefore, studying the equity of TCM health resource allocation and forecasting the development of the 14th Five-Year Plan will help to further improve the supply of TCM health resources and promote the development of TCM health services.

With the change of the disease spectrum, the incidence rate of sub-health population and chronic diseases has greatly increased, and the field of disease prevention and rehabilitation is becoming more and more important. TCM plays an important role in the prevention of diseases, treatment of major diseases, and rehabilitation of diseases, which has opened up a wide space for the development of TCM. At present, the relevant research on the health resources of TCM mainly includes the analysis of health resources and utilization in TCM hospitals [8], the analysis of regional differences in health resources of county-level TCM hospitals [9], and the analysis of the efficiency of the allocation of health resources of TCM [10]. The analysis on the equity and development prediction of TCM health resources [11], such as the number of beds and licensed (assistant) doctors of TCM, is relatively small, and the research content needs to be further deepened.

## Materials and methods

### Data sources

The data of TCM health resources were taken from the China Health Statistics Yearbook in 2017–2021, and the number of permanent residents, geographical area and regional gross domestic product(GDP) were taken from the China Statistics Yearbook in 2017–2021, which covered 31 provinces, autonomous regions and municipalities (excluding Hong Kong, Macao and Taiwan). This is a longitudinal study to analyze the equity of health resource allocation of TCM in China from 2016 to 2020 and to predict the development of the 14th Five-Year Plan period.

### Region division

According to the new situation of coordinated development of China’s economic and social regions, and in accordance with the division principle of revitalizing the Northeast region, Eastern region taking the lead in developing, Central region rising and Western region development, the Chinese Mainland (excluding Hong Kong, Macao and Taiwan) is divided into Northeast region (Heilongjiang, Jilin and Liaoning), Eastern region (Beijing, Tianjin, Hebei, Shandong, Jiangsu, Shanghai, Zhejiang, Fujian, Guangdong and Hainan), Central region (Shanxi, Henan, Hunan, Hubei, Jiangxi and Anhui) and Western region (Chongqing, Sichuan, Guangxi, Yunnan, Guizhou, Shaanxi, Gansu, Inner Mongolia, Qinghai, Ningxia, Xinjiang and Tibet).

### Indicators

According to previous related studies, physical resources and human resources are important indicators of health services, the number of TCM medical institutions, the number of TCM beds, practitioners (assistants) of TCM and Chinese pharmacists from 2016 to 2020 were selected as evaluation indicators. The number of TCM medical institutions and beds represent physical resources, and practitioners (assistants) of TCM and Chinese pharmacists represent human resources.

### Data analysis

Descriptive analysis, Concentration index(CI), Theil index(T) and Health resource agglomeration degree(HRAD) were used to evaluate the equity of health resource allocation of TCM. Grey prediction model GM (1,1) was used to predict the development of health resources of TCM during the 14th Five-Year Plan period.

The Concentration index is an important indicator recommended by the World Bank to evaluate the equity of health services among regions with different levels of economic development. The value range is—1~1, and the absolute value means that the greater the equity, the more unfair [12]. A positive value indicates that health resources tend to regions with higher economic development level, while a negative value indicates that health resources tend to regions with lower economic development level. The calculation formula is:

$$CI=1-\sum_{i=1}^{n}\left(Y_{i-1}+Y_{i})(X_{i}+X_{i-1}\right)$$

Where, n is the number of regions, X_0_ = 0, Y_0_ = 0, Xi is the cumulative percentage of the population in each region ranked from low to high by GDP per capita, and Yi refers to the cumulative percentage of health resources.

Theil index is an index used to measure the equity of health resources [13]. It is also a good decomposition index, which can well reflect the differences within and between regions and the contribution of each part of the difference to the total difference [14]. The smaller the Thiel index, the better the equity. The contribution rate refers to the share of the differences within and between regions in the total differences [15]. The greater the contribution rate, the greater the impact of inequity. The calculation formula is:

$$T=\sum_{i=1}^{n}P_{i}\log\frac{P_{i}}{Y_{i}}$$

Where, n is the number of regions, Pi is the proportion of population in the ith region to the total population, and Yi is the proportion of health resources in the ith region to the total health resources.

The HRAD is usually combined with the Population agglomeration degree(PAD) to comprehensively evaluate the number of health services population and geographical area. The HRAD is the proportion of TCM health resources gathered on 1% of China’s land area in a region, and the PAD is the proportion of people gathered on 1% of China’s land area in a region [16]. The evaluation standard is that HRAD less than 1 indicates that the equity of geographical allocation of TCM health resources is insufficient, while HRAD greater than 1 indicates that the equity of geographical allocation of TCM health resources is good. HRAD/PAD greater than 1 indicates that the equity of TCM health resources is better than that of the agglomeration population, and less than 1 indicates that TCM health resources cannot meet the medical needs of the agglomeration population [17]. The calculation formula is:

$${\mathrm{HRAD}}_{i}=\frac{(\frac{{\mathrm{HR}}_{i}}{{\mathrm{HR}}_{n}})X100\%}{(\frac{A_{i}}{A_{n}})X100\%}$$

Where, HRi is the number of TCM health resources in region i, HRn is the total amount of TCM health resources, Ai is the land area in region i, and An is the total land area. PAD is calculated by replacing the number of health resources with the number of population.

The grey prediction model GM (1,1) is to build a continuous differential equation with time as a variable, and determine the parameters in the equation by mathematical methods to achieve prediction [18]. It has the characteristics of small amount of original sample data, high prediction accuracy, and can be used for long-term prediction. In recent years, it has been widely used in the field of medicine and health. The construction method of GM (1,1) model is to accumulate the original data series, construct the accumulation matrix and constant vector, solve the grey parameters a and u by the least square method, and finally establish the time response equation, that is, the specific calculation formula of GM (1,1) model (k is the time series). The calculation formula is:

$${\hat{X}}^{\left(1\right)}(k+1)=(X^{\left(0\right)}(1)-\frac{u}{a})e^{-ak}+\frac{u}{a}$$

The posterior error ratio (C value) and small error probability (P value) are used to test the accuracy of the prediction model. When P is greater than 0.95 and C is less than 0.35, the prediction model is reliable and can be used for prediction.

### Ethics statement

The data of the Statistical Yearbook are publicly available. Ethical approval is not needed because there is no secondary data for any personal information.

## Results

### Basic information of TCM health resources

The health resources of TCM have been well developed. The number of TCM medical institutions, TCM beds, practitioners (assistants) of TCM and Chinese pharmacists has increased from 49 527, 1 033 547, 481 590, 116 622 in 2016 to 72 355, 1 432 900, 682 770, 131 163 in 2020 (Table 1).

**Table 1:** Basic information of TCM health resources.

Year	TCM medical institutions	TCM beds	Practitioners (assistants) of TCM	Chinese pharmacists
2016	49 527	1 033 547	481 590	116 622
2017	54 243	1 135 525	527 047	120 302
2018	60 738	1 234 237	575 454	123 913
2019	65 809	1 328 752	624 783	127 154
2020	72 355	1 432 900	682 770	131 163

### Concentration index of TCM health resources

From 2016 to 2020, the Concentration index of the number of TCM medical institutions and TCM beds is negative, indicating that the number of TCM medical institutions and TCM beds tends to the regions with low economic development level. From 2016 to 2020, the Concentration index of practitioners (assistants) of TCM and Chinese pharmacists is positive, indicating that the allocation of practitioners (assistants) of TCM and Chinese pharmacists tends to the regions with high economic development level. The absolute number of the Concentration index of TCM beds shows an increasing trend, indicating that the equity of the number of TCM beds has gradually deteriorated (Table 2).

**Table 2:** Concentration index of TCM health resources.

Year	TCM medical institutions	TCM beds	Practitioners (assistants) of TCM	Chinese pharmacists
2016	-0.041 0	-0.021 2	0.032 9	0.116 8
2017	-0.039 7	-0.023 6	0.040 2	0.119 3
2018	-0.027 9	-0.028 0	0.041 0	0.115 2
2019	-0.048 3	-0.041 9	0.018 4	0.094 8
2020	-0.051 2	-0.064 0	0.004 6	0.082 6

### Theil index of TCM health resources by population

From the perspective of the Theil index allocated by population, the number of TCM medical institutions, practitioners (assistants) of TCM and Chinese pharmacists showed a downward trend from 2016 to 2020, indicating that the equity of the number of TCM medical institutions, practitioners (assistants) of TCM and Chinese pharmacists gradually improved; The Theil index of the number of TCM beds showed an upward trend, indicating that the equity of the number of TCM beds gradually worsened. The Theil index for the number of TCM medical institutions, practitioners (assistants) of TCM and Chinese pharmacists between regions was larger than within regions, indicating that the inequity mainly came from between regions. The Theil index for the number of TCM beds within regions was larger than between regions, indicating that the inequity mainly comes from within regions (Table 3).

**Table 3:** Theil index of TCM health resources by population.

Year	TCM medical institutions			TCM beds			Practitioners (assistants) of TCM			Chinese pharmacists		
	Theil index	Within region	Between region	Theil index	Within region	Between region	Theil index	Within region	Between region	Theil index	Within region	Between region
2016	0.054 2	0.020 5	0.033 7	0.008 7	0.004 6	0.004 1	0.015 7	0.003 0	0.012 7	0.023 1	0.005 9	0.017 2
2017	0.049 9	0.018 4	0.031 4	0.009 4	0.005 0	0.004 4	0.014 9	0.002 7	0.012 2	0.023 2	0.005 7	0.017 5
2018	0.047 8	0.017 0	0.030 8	0.010 3	0.005 8	0.004 5	0.014 4	0.002 4	0.012 0	0.021 9	0.005 3	0.016 6
2019	0.043 4	0.014 8	0.028 6	0.011 2	0.006 3	0.005 0	0.013 4	0.001 9	0.011 5	0.022 4	0.005 2	0.017 2
2020	0.037 6	0.014 8	0.022 8	0.012 8	0.009 0	0.003 9	0.011 0	0.002 6	0.008 4	0.020 0	0.005 4	0.014 5

### Theil index contribution rate of TCM health resources by population

From the perspective of the contribution rate of the Theil index by population, the between regions contribution rate of the number of TCM medical institutions to the Theil index has exceeded 60%, and the between regions contribution rate of practitioners (assistants) of TCM and Chinese pharmacists to the Theil index has exceeded 72%, indicating that the inequity of the number of TCM medical institutions, practitioners (assistants) of TCM and Chinese pharmacists mainly comes from the between regions. The within regions contribution rate of the number of TCM beds to the Theil index has exceeded 52%, indicating that the inequity of the allocation of TCM beds mainly comes from the within regions (Table 4).

**Table 4:** Theil index contribution rate of TCM health resources by population.

Year	TCM medical institutions		TCM beds		Practitioners (assistants) of TCM		Chinese pharmacists	
	Within region	Between region	Within region	Between region	Within region	Between region	Within region	Between region
2016	37.83	62.17	52.88	47.12	18.89	81.11	25.69	74.31
2017	36.97	63.03	53.62	46.38	17.92	82.08	24.56	75.44
2018	35.59	64.41	56.31	43.69	16.53	83.47	24.11	75.89
2019	34.08	65.92	55.64	44.36	14.04	85.96	23.29	76.71
2020	39.32	60.98	69.89	30.11	24.04	75.96	27.31	72.69

### Theil index of TCM health resources by geography

From 2016 to 2020, the Theil index of the number of TCM medical institutions, TCM beds, practitioners (assistants) of TCM and Chinese pharmacists by geography is greater than that by population, indicating that the equity of health resources of TCM by population is better than that by geography. The Theil index of the number of TCM medical institutions by geography shows an increasing trend, indicating that the equity has become worse. The Theil index of the number of TCM beds, practitioners (assistants) of TCM and Chinese pharmacists by geography shows a downward trend, indicating that the equity is gradually improving. The number of TCM medical institutions, TCM beds, practitioners (assistants) of TCM and Chinese pharmacists within region have a higher Theil index than that between regions, indicating that the inequity of TCM health resources by geography mainly comes from within region (Table 5).

**Table 5:** Theil index of TCM health resources by geography.

Year	TCM medical institutions			TCM beds			Practitioners (assistants) of TCM			Chinese pharmacists		
	Theil index	Within region	Between region	Theil index	Within region	Between region	Theil index	Within region	Between region	Theil index	Within region	Between region
2016	0.413 4	0.336 5	0.076 9	0.460 9	0.375 2	0.085 7	0.480 1	0.385 9	0.094 2	0.523 0	0.423 2	0.099 8
2017	0.412 3	0.336 9	0.075 4	0.453 2	0.369 6	0.083 6	0.476 7	0.383 5	0.093 3	0.517 2	0.418 8	0.098 4
2018	0.422 1	0.344 6	0.077 5	0.450 9	0.367 6	0.083 3	0.467 1	0.376 4	0.090 7	0.511 5	0.414 3	0.097 2
2019	0.432 3	0.351 7	0.080 7	0.446 5	0.363 5	0.083 0	0.470 2	0.377 9	0.092 3	0.506 1	0.409 3	0.096 8
2020	0.444 8	0.362 2	0.082 6	0.443 0	0.361 0	0.082 1	0.469 0	0.377 4	0.091 6	0.505 8	0.408 7	0.097 0

### Theil index contribution rate of TCM health resources by geography

The number of TCM medical institutions, TCM beds, practitioners (assistants) of TCM and Chinese pharmacists within region contributed more than 80% to the Thiel index, indicating that the inequity of TCM health resources by geography mainly comes from within region (Table 6).

**Table 6:** Theil index contribution rate of TCM health resources by geography.

Year	TCM medical institutions		TCM beds		Practitioners (assistants) of TCM		Chinese pharmacists	
	Within region	Between region	Within region	Between region	Within region	Between region	Within region	Between region
2016	81.41	18.59	81.41	18.59	80.38	19.62	80.92	19.08
2017	81.72	18.28	81.55	18.45	80.43	19.57	80.97	19.03
2018	81.63	18.37	81.53	18.47	80.58	19.42	81.00	19.00
2019	81.34	18.66	81.41	18.59	80.37	19.63	80.88	19.12
2020	81.43	18.57	81.48	18.52	80.47	19.53	80.81	19.19

### HRAD of TCM health resources

From the perspective of PAD, China’s population is mainly agglomerated in the Eastern and Central regions, while the population in the Northeast and Western regions is relatively small.

From the perspective of HRAD, the HRAD in the Eastern and Central regions is greater than 1, indicating that the equity of TCM health resource allocation in the Eastern and Central regions is good. The HRAD in Northeast region (excluding the number of TCM medical institutions) and Western region is less than 1, indicating that the equity of health resources allocation in Northeast (excluding the number of TCM medical institutions) and Western regions is insufficient.

From the perspective of HRAD/PAD, the HRAD/PAD of practitioners (assistants) of TCM in the Northeast region is less than 1, the HRAD/PAD of the number of TCM medical institutions and TCM beds in East region is less than 1, and the HRAD/PAD of health resources in the Central region (excluding the number of TCM beds in 2020) is less than 1, indicating that the health resources of TCM in these regions cannot meet the medical needs of the agglomerated population. The HRAD/PAD of the number of TCM medical institutions, TCM beds and practitioners (assistants) of TCM in the Western region are greater than 1, indicating that the health resources in the Western region are fairly fair compared with the agglomerated population (Table 7).

**Table 7:** HRAD of TCM health resources.

Year	Region	PAD	HRAD				HRAD/PAD			
			TCM medical institutions	TCM beds	practitioners (assistants) of TCM	Chinese pharmacists	TCM medical institutions	TCM beds	practitioners (assistants) of TCM	Chinese pharmacists
2016	Northeast region	0.949 3	1.215 7	0.896 5	0.822 4	0.966 4	1.280 7	0.944 4	0.866 3	1.018 0
	East region	3.908 4	3.202 2	3.457 9	4.057 0	4.233 3	0.819 3	0.884 7	1.038 0	1.083 1
	Central region	2.462 7	1.803 5	2.445 6	2.106 7	2.373 6	0.732 3	0.993 1	0.855 4	0.963 8
	West region	0.381 6	0.548 2	0.452 7	0.430 1	0.348 3	1.436 5	1.186 2	1.127 0	0.912 6
2017	Northeast region	0.940 5	1.198 3	0.896 9	0.826 9	0.982 5	1.274 2	0.953 7	0.879 3	1.044 7
	East region	3.914 7	3.371 3	3.436 1	4.098 5	4.316 2	0.861 2	0.877 7	1.046 9	1.102 6
	Central region	2.460 3	1.769 2	2.432 7	2.093 7	2.287 3	0.719 1	0.988 8	0.851 0	0.929 6
	West region	0.382 1	0.532 1	0.457 6	0.425 8	0.348 0	1.392 4	1.197 5	1.114 3	0.910 8
2018	Northeast region	0.931 6	1.217 3	0.904 5	0.832 3	0.980 2	1.306 7	0.970 9	0.893 4	1.052 1
	East region	3.919 9	3.552 2	3.371 7	4.145 2	4.327 8	0.906 2	0.860 1	1.057 5	1.104 0
	Central region	2.459 9	1.697 5	2.444 8	2.068 8	2.236 9	0.690 1	0.993 9	0.841 0	0.909 3
	West region	0.382 5	0.515 8	0.463 8	0.422 5	0.354 4	1.348 3	1.212 3	1.104 5	0.926 4
2019	Northeast region	0.923 1	1.151 6	0.875 6	0.808 4	0.959 4	1.247 4	0.948 5	0.875 7	1.039 3
	East region	3.929 6	3.660 3	3.324 4	4.170 5	4.374 4	0.931 5	0.846 0	1.061 3	1.113 2
	Central region	2.456 0	1.696 7	2.430 5	2.041 9	2.178 9	0.690 8	0.989 6	0.831 4	0.887 2
	West region	0.382 8	0.508 7	0.475 9	0.425 9	0.359 2	1.328 9	1.243 2	1.112 7	0.938 4
2020	Northeast region	0.839 0	1.156 6	0.853 0	0.785 5	0.937 5	1.378 6	1.016 8	0.936 3	1.117 5
	East region	4.072 5	3.660 6	3.276 3	4.126 9	4.391 9	0.898 9	0.804 5	1.013 3	1.078 4
	Central region	2.394 7	1.802 1	2.508 2	2.134 0	2.190 7	0.752 5	1.047 4	0.891 1	0.914 8
	West region	0.382 2	0.492 0	0.473 3	0.420 6	0.357 6	1.287 2	1.238 4	1.100 5	0.935 5

### Prediction model and test results of TCM health resources

Taking the data of health resources of TCM from 2016 to 2020 as the original data sequence, a prediction model for the number of TCM medical institutions, TCM beds, practitioners (assistants) of TCM and Chinese pharmacists is established according to the calculation steps of the gray prediction model GM (1,1). The posterior error ratio (C value) and small error probability (P value) are used to test the accuracy of the prediction model. In the prediction model, P is greater than 0.95, C is less than 0.35, and the accuracy level is level 1, whinch indicates that the prediction fitting effect of each model is good, the model prediction is accurate and reliable, and can be used for extrapolation prediction (Table 8).

**Table 8:** Prediction model and test results of TCM health resources.

Predictive Indicators	Parameter Value	Prediction Model	C	P	Accuracy Level
TCM medical institutions	a = -0.094,u = 47 476.775	554 599.074 5e^0.094k^-505 072.074 5	0.002	1.000	Level 1
TCM beds	a = -0.077,u = 1 015 606.212	14 223 238.06e^0.077k^-13 189 691.060	0.000	1.000	Level 1
Practitioners (assistants) of TCM	a = -0.086,u = 463 223.819	5 867 913.477e^0.086k^-5 386 323.477	0.000	1.000	Level 1
Chinese pharmacists	a = -0.029,u = 115 265.502	4 091 294.483e^0.029k^-3 974 672.483	0.001	1.000	Level 1

### Prediction results of TCM health resources from 2016 to 2025

According to the prediction, the predicted values of four indicators, including the number of TCM medical institutions, from 2016 to 2020 are consistent with the actual values, and the relative error is within 1.201%, indicating that the prediction is reasonable. From 2021–2025, TCM health resources will continue to develop to better protect people’s health in an all-round and full-cycle. According to the prediction, the number of TCM medical institutions, TCM beds, practitioners (assistants) of TCM and Chinese pharmacists will reach 115 651.623, 2 105 577.720, 1 047 275.131 and 151 141.836 by 2025 (Table 9).

**Table 9:** Prediction results of TCM health resources from 2016 to 2025.

Year	TCM medical institutions		TCM beds		Practitioners (assistants) of TCM		Chinese pharmacists	
	Predictive value	Relative error (%)	Predictive value	Relative error (%)	Predictive value	Relative error (%)	Predictive value	Relative error (%)
2016	49 527.000	0.000	1 033 547.000	0.000	481 590.000	0.000	116 622.000	0.000
2017	54 639.487	0.731	1 138 268.076	0.242	526 877.471	0.032	120 300.367	0.001
2018	60 008.428	1.201	1 229 236.354	0.405	574 121.121	0.232	123 781.764	0.106
2019	65 904.929	0.146	1 327 474.649	0.096	625 600.978	0.131	127 363.911	0.165
2020	72 380.826	0.036	1 433 563.966	0.046	681 696.893	0.157	131 049.722	0.086
2021	79 493.052	—	1 548 131.745	—	742 822.773	—	134 842.198	—
2022	87 304.135	—	1 671 855.569	—	809 429.642	—	138 744.425	—
2023	95 882.744	—	1 805 467.172	—	882 008.966	—	142 759.579	—
2024	105 304.297	—	1 949 756.767	—	961 096.278	—	146 890.928	—
2025	115 651.623	—	2 105 577.720	—	1 047 275.131	—	151 141.836	—

## Discussion

During the "13th Five-Year Plan" period, the policy environment for the development of TCM has been further optimized, the support of health policies has been further increased, and the health resources of TCM have been rapidly increased, making important contributions to meeting the health needs of the people. According to the prediction results, the number of TCM medical institutions, TCM beds, practitioners (assistants) of TCM and Chinese pharmacists will reach 115 651.623, 2 105 577.720, 1 047 275.131 and 151 141.836 by 2025. The allocation of health resources of TCM has become more abundant, and the service capacity of TCM has been enhanced, providing strong support for the all-round protection of people’s health.

From the perspective of Concentration index, the number of TCM medical institutions and TCM beds tends to the regions with low economic development level, and the number of practitioners (assistants) of TCM and Chinese pharmacists tends to the regions with high economic development level, which indicates that the allocation of different health resources has been differentiated, health material resources tend to be more subject to policy regulation by administrative departments, and health human resources tend to flow to areas with high economic development level. Generally speaking, regions with a higher level of economic development have better financial income, so they can invest more in the development of TCM [19]. Regions with a high level of economic development level have natural attraction for health human resources, which leads to the flow of health human resources from west to east and from rural areas to cities [20]. In addition to the influence of the level of economic development, policy regulation is also an important factor [21]. The number of TCM medical institutions and TCM beds and other health material resources are obviously affected by the policy regulation, which can promote the rapid construction of regions with low economic development level through the planning and construction of TCM, increasing financial investment and other measures, thus improving the allocation level of regions with weak allocation of TCM health resources. On the contrary, health human resources such as practitioners (assistants) of TCM and Chinese pharmacists are less affected by policy regulation, and more vulnerable to factors such as more competitive salary and treatment, more room for career advancement, and more convenient social transportation conditions in regions with higher economic development levels, so they are more willing to move to regions with higher economic development levels [22]. Regions with higher economic development level have better medical care awareness and higher medical service payment capacity, and the frequency of medical service utilization will be significantly higher than that of regions with lower economic development level, thus forming a fully developed and mature medical market, further promoting the flow of health resources to these regions with higher economic development level [23]. Therefore, all regions should rely on their own regional advantages to actively develop the regional economy, make full use of the "the Belt and Road" and other policies to go out and introduce, vigorously develop the social economy, and make the foundation for the development of TCM bigger and stronger. In terms of the number of TCM medical institutions, TCM beds and other policies that play an obvious role in regulation, we should strengthen the regulation of regions with low economic development level, strengthen the construction of TCM health resources in remote and backward regions, and promote everyone’s access to TCM health services [24]. In terms of the obvious market role of practitioners (assistants) of TCM and Chinese pharmacists, we should further optimize the market order and promote the high-quality development of TCM health resources.

From the perspective of the Theil index, the equity of the health resources allocation of TCM by population is better than that by geography. This shows that the equity of the allocation of TCM health resources by geography is poor, which is not conducive to the people in remote and backward areas enjoying the same TCM services. For a long time, health resources have been based on the amount of health resources per thousand people, thus ignoring the impact of geographical factors on health resources [25]. In remote and backward areas, the population is small, the geographical area is large, the allocation of health resources is poor, the radius of health services is too large, and the accessibility of health services is poor [26]. With the increasingly convenient transportation and the implementation of the reimbursement policies for medical insurance in other places, more and more patients prefer to choose medical institutions in areas with higher levels of TCM services for medical treatment, resulting in fewer visits to TCM service institutions in grassroots areas, rural areas and other areas with weak TCM health resources, thus increasing the geographical inequity of TCM health resources.

From the decomposition of the Theil index, the inequity of the number of TCM medical institutions, practitioners (assistants) of TCM and Chinese pharmacists by population mainly comes from between regions, and the inequity of TCM health resources by geography mainly comes from within regions. In terms of population allocation, China’s population is mainly concentrated in the Eastern and Central regions, while the population in the Northeast and Western regions is relatively small. In addition, the superposition effect of the relatively high level of economic development in the Eastern and Central regions leads to the obvious difference in the allocation of TCM health resources between regions [27]. In terms of geographical allocation, there are large differences in the allocation of TCM health resources within regions. On the one hand, the geographical location and health policies of each region are different, which leads to the different allocation of health resources. The health resources in regions with good geographical advantages and policy emphasis develop rapidly, while the health resources in regions with remote geographical location and policy neglect develop slowly [28]. On the other hand, TCM health resources are subject to different degrees of "siphoning effect". Regional central cities siphon off surrounding cities, provincial capital cities siphon off other cities in the region, and urban areas siphon off rural areas, resulting in large disparities in the geographical allocation of health resources within regions. Therefore, the allocation of TCM health resources should comprehensively consider population and geographical factors [29], focus on the degree of equity in terms of geographical distribution, and focus on strengthening the accessibility of TCM services in remote and backward areas. We should strengthen the integration of health resources of TCM, make overall plans for economically developed and underdeveloped areas, promote the sinking of health resources of TCM, promote the rational flow of health resources of TCM through targeted support, counterpart support, targeted training and other ways, and improve the ability and level of TCM services in areas with insufficient resources [30].

From the perspective of HRAD, TCM health resources are mainly agglomerated in the Eastern and Central regions, and relatively few in the Northeast and Western regions. From the perspective of HRAD/PAD, the Central region cannot meet the medical needs of the agglomerated population, and the Western region has better equity compared with the agglomerated population. This shows that the health resources of TCM in the four regions are quite different, and there is a contradiction between equity and actual medical needs in the Central and Western regions. The health resources of TCM are mainly agglomerated in the Eastern and Central regions, where the population is relatively agglomerated, the social and economic development level is relatively high, and the transportation conditions are relatively convenient, so the health resources are relatively agglomerated. The economic development level of the Northeast and Western regions is relatively low, the geographical area is relatively wide, the radius distance of TCM service is large, and the service cost is relatively high, resulting in relatively few TCM health resources in these two regions. The four regions have large differences in TCM health resources, which limits the realization of the goal of everyone enjoying TCM health services. From the actual situation of the four regions, the geographical distribution of TCM health resources in the Central region is relatively agglomerated, but it is insufficient compared with the agglomerated population; the geographical distribution of health resources of TCM in the Western region is relatively insufficient, but relative to the agglomerated population, it appears to be excessive; this coexistence of surplus and shortage in agglomerated population and geographical distribution is also reflected in the Eastern and Northeast regions, that is, the equity is in contradiction with the actual medical needs. In the case of the contradiction between the equity and the actual medical needs, it is easy to have a higher equity of geographical allocation in the actual use of health services, but the health resources are insufficient relative to the agglomerated population; the equity of population allocation is excessive, but the equity of health resources relative to geography is poor, which greatly affects the equity and accessibility of TCM health services. First of all, we should strengthen the allocation of TCM health resources in weak areas. Based on the overall growth of TCM health resources, all regions should improve the allocation of TCM health resources through policy support [31], financial subsidies, inheritance and innovation of TCM talents, etc. Second, in regions with agglomerated population, we should focus on the equity of TCM health resources relative to the agglomerated population, and constantly improve the quality and level of TCM services; in regions with few TCM health resources, we should focus on the equity of TCM health resources relative to geography, and focus on strengthening the coverage of TCM services [32]. Third, it is necessary to strengthen the vertical integration of TCM health resources, strengthen the support from provincial and municipal large hospitals to county-level hospitals and grassroots medical institutions, select appropriate technologies of TCM for promotion, and promote high-quality TCM resources to benefit the people.

## Limitations

Although we used the CI, T, HRAD and gray prediction model GM(1,1) to analyze the equity of TCM health resource allocation in China and its development during the 14th Five-Year Plan period, there are still some limitations in this study. First, the evaluation indices were selected based on previous related studies and are the evaluation indices usually used in similar studies. Second, equity and efficiency are important elements of health resource allocation, and this study did not analyze the efficiency of TCM health resource allocation in China. Third, this study mainly analyzed from the perspective of health resource allocation and did not take into account the actual health status and health service needs of different regions.

## Conclusion

In the context of the inheritance and innovative development of TCM, in this study, the Chinese Mainland (excluding Hong Kong, Macao and Taiwan) was divided into Northeast region, Eastern region, Central region and Western region. Descriptive analysis, Concentration index, Theil index and Health resource agglomeration degree were used to evaluate the equity of TCM health resource allocation, and the gray prediction model GM (1,1) was used to predict the development of TCM health resources during the 14th Five-Year Plan period. From the perspective of the Concentration index, the number of TCM medical institutions and TCM beds tends to the regions with low economic development level, and the number of practitioners (assistants) of TCM and Chinese pharmacists tends to the regions with high economic development level. From the perspective of the Theil index, the equity of the allocation of TCM health resources by population is better than that by geography. From the decomposition of the Theil index, the inequity of the number of TCM medical institutions, practitioners (assistants) of TCM and Chinese pharmacists by population mainly comes from between regions, and the inequity of TCM health resources by geography mainly comes from within regions. From the perspective of HRAD, TCM health resources are mainly agglomerated in the Eastern and Central regions, and relatively few in the Northeast and Western regions. From the perspective of HRAD/PAD, the Central region can not meet the medical needs of the agglomerated population, and the Western region has better equity compared with the agglomerated population. According to the prediction results, the number of TCM medical institutions, TCM beds, practitioners (assistants) of TCM and Chinese pharmacists will reach 115 651.623, 2 105 577.720, 1 047 275.131 and 151 141.836 by 2025. In order to promote the construction of the service system of TCM, the health resources of TCM should comprehensively take into account demographic and geographical factors, strengthen the construction of health resources of TCM in weak areas, especially in the Northeast and Western regions, select appropriate technologies of TCM for promotion, and ensure people’s health in an all-round and full-cycle.

## Supporting information

S1 Data Data of traditional Chinese medicine health resources in 31 provinces, autonomous regions and municipalities of China, 2016–2020. (XLSX)

## References

[1] Xu Wen-Jie, Wang Ling-Tai, Zhao Zhi-Ping, Zhu Li-Ming, Zu Liang-Hua, Zhang Qi, et al. Prospects of a comprehensive evaluation system for traditional Chinese medicine services[J]. Journal of Integrative Medicine. 2017,15(6):426–432. doi: 10.1016/S2095-4964(17)60364-9 29103411

[2] The State Council of China. The Notice of the State Council on the Printing and Distributing the Strategic Planning Outline for the Development of Traditional Chinese Medicine[EB/OL]. https://www.gov.cn/zhengce/zhengceku/2016-02/26/content_5046678.htm. Accessed February 26, 2016.

[3] General Office of the State Council of China. The Notice of the General Office of the State Council on the Printing and Distribution of the Fourteenth Five-Year Plan for the Development of Traditional Chinese Medicine[EB/OL]. https://www.gov.cn/gongbao/content/2022/content_5686029.htm. Accessed March 3, 2022.

[4] Tao Wenjuan, Zeng Zhi, Dang Haixia, Lu Bingqing, Chuong Linh, Yue Dahai, et al. Towards universal health coverage: lessons from 10 years of healthcare reform in China[J]. BMJ Global Health. 2020,5(3):e002086. doi: 10.1136/bmjgh-2019-002086 PMC7103824 32257400

[5] Dai Guolin, Li Ruifeng, Ma Shuang. Research on the equity of health resource allocation in TCM hospitals in China based on the Gini coefficient and agglomeration degree: 2009–2018[J]. International Journal for Equity in Health. 2022,21(1):145. doi: 10.1186/s12939-022-01749-7 36199086 PMC9534739

[6] Yu Huimin, Yu Shuangyan, He Da, Lu Yuanan. Equity analysis of Chinese physician allocation based on Gini coefficient and Theil index[J]. BMC Health Services Research. 2021,21(1):455. 33980223 10.1186/s12913-021-06348-w PMC8115393

[7] Päivi Rouvinen-Wilenius Jussi Ahokas, Kiukas Vertti, Mervi Aalto-Kallio. Finnish NGOs promoting health equity in the context of welfare economy[J]. Health Promotion International. 2019,34(4):648–657. 29659821 10.1093/heapro/day022

[8] Shi Xuefeng, Zhu Dawei, Nicholas Stephen, Hong Baolin, Man Xiaowei, He Ping. Is Traditional Chinese Medicine "Mainstream" in China? Trends in Traditional Chinese Medicine Health Resources and Their Utilization in Traditional Chinese Medicine Hospitals from 2004 to 2016[J]. Evidence-Based Complementary and Alternative Medicine. 2020:9313491. 10.1155/2020/9313491 PMC7281804 32595750

[9] Zhu Dawei, Shi Xuefeng, Nicholas Stephen, He Ping. Regional disparities in health care resources in traditional Chinese medicine county hospitals in China[J]. PLoS One. 2020,15(1):e0227956. 31961912 10.1371/journal.pone.0227956 PMC6974170

[10] Li Zhengjun, Yang Lili, Tang Shaoliang, Bian Yaoyao. Equity and Efficiency of Health Resource Allocation of Chinese Medicine in Mainland China: 2013–2017[J]. Frontiers in Public Health. 2020,8:579269. 33384979 10.3389/fpubh.2020.579269 PMC7769806

[11] Xu Rixiang, Mu Tingyu, Liu Yulian, Ye Yaping, Xu Caiming. Trends in the disparities and equity of the distribution of traditional Chinese medicine health resources in China from 2010 to 2020[J]. PLoS One. 2022,17(10):e0275712. 36215249 10.1371/journal.pone.0275712 PMC9550081

[12] Li Xiao-Mei, Kou Jing, Yu Zhen, Xiao Yuan-Yuan, Meng Qiong, He Li-Ping. Health Equity of Rural Residents in Southwest China[J]. Frontiers in Public Health. 2021,9:611583. 33834014 10.3389/fpubh.2021.611583 PMC8021781

[13] Pu Lida. Fairness of the Distribution of Public Medical and Health Resources[J]. Frontiers in Public Health. 2021,9:768728. 34858935 10.3389/fpubh.2021.768728 PMC8631734

[14] Yang Yong, Nicholas Stephen, Maitland Elizabeth, Huang Zhengwei, Chen Xiaoping, Ma Yong, et al. An equity evaluation in stroke inpatients in regard to medical costs in China: a nationwide study[J]. BMC Health Services Research. 2021,21(1):425. 33952266 10.1186/s12913-021-06436-x PMC8097888

[15] Yang Le, Cheng Jingmin. Analysis of inequality in the distribution of general practitioners in China: evidence from 2012 to 2018[J]. Primary Health Care Research & Development. 2022,23:e59. 36117274 10.1017/S1463423622000408 PMC9532852

[16] Wang Yueyue, Li Yuyang, Qin Shangren, Kong Yuanfeng, Yu Xiyang, Guo Keqiang, et al. The disequilibrium in the distribution of the primary health workforce among eight economic regions and between rural and urban areas in China[J]. International Journal for Equity in Health. 2020,19(1):28. 32102655 10.1186/s12939-020-1139-3 PMC7045560

[17] Yu Qianqian, Yin Wenqiang, Huang Dongmei, Sun Kui, Chen Zhongming, Guo Hongwei, et al. Trend and equity of general practitioners’ allocation in China based on the data from 2012–2017[J]. Human Resources for Health. 2021,19(1):20. 33588888 10.1186/s12960-021-00561-8 PMC7885472

[18] Cheng Tingting, Bai Yu, Sun Xianzhi, Ji Yuchen, Zhang Fan, Li Xiaofeng. Epidemiological analysis of varicella in Dalian from 2009 to 2019 and application of three kinds of model in prediction prevalence of varicella[J]. BMC Public Health. 2022,22(1):678. 35392857 10.1186/s12889-022-12898-3 PMC8991558

[19] Li Qian, Wei Jianjun, Jiang Fengchang, Zhou Guixiang, Jiang Rilei, Chen Meijuan, et al. Equity and efficiency of health care resource allocation in Jiangsu Province, China[J]. International Journal for Equity in Health. 2020,19(1):211. 33246458 10.1186/s12939-020-01320-2 PMC7694921

[20] Yao Honghui, Zhan Chaohong, Sha Xinping. Current situation and distribution equality of public health resource in China[J]. Archives of Public Health. 2020,78:86. 32983449 10.1186/s13690-020-00474-3 PMC7507592

[21] Woldemichael Abraha, Takian Amirhossein, Ali Akbari Sari Alireza Olyaeemanesh. Inequalities in healthcare resources and outcomes threatening sustainable health development in Ethiopia: panel data analysis[J]. BMJ Open. 2019,9(1):e022923. 10.1136/bmjopen-2018-022923 PMC6359736 30705237

[22] Lu Liming, Zeng Jingchun. Inequalities in the geographic distribution of hospital beds and doctors in traditional Chinese medicine from 2004 to 2014[J]. International Journal for Equity in Health. 2018,17(1):165. 30419919 10.1186/s12939-018-0882-1 PMC6233493

[23] Cai Yi, Mao Zongfu, Xu Bruce, Wu Bei. Factors associated with traditional Chinese medicine utilization among urban community health centers in Hubei Province of China[J]. Asia-Pacific Journal of Public Health.2015,27(2):NP2489–97. 23858517 10.1177/1010539513491415

[24] Yang Le, Wang Hongman, Xue Lei. What about the health workforce distribution in rural China? An assessment based on eight-year data[J]. Rural and Remote Health.2019,19(3):4978. 31340655 10.22605/RRH4978

[25] Jin Jian, Wang Jianxiang, Ma Xiaoyi, Wang Yuding, Li Renyong. Equality of Medical Health Resource Allocation in China Based on the Gini Coefficient Method[J]. Iranian Journal of Public Health. 2015,44(4):445–57. 26056663 PMC4441957

[26] Cao Wen-Rui, Shakya Prabin, Karmacharya Biraj, Dong Roman Xu Yuan-Tao Hao, Lai Ying-Si. Equity of geographical access to public health facilities in Nepal[J]. BMJ Global Health. 2021,6(10):e006786. 10.1136/bmjgh-2021-006786 PMC8552161 34706879

[27] Liu Tao, Li Jixia, Chen Juan, Yang Shaolei. Regional Differences and Influencing Factors of Allocation Efficiency of Rural Public Health Resources in China[J]. Healthcare (Basel). 2020,8(3):270. 32823864 10.3390/healthcare8030270 PMC7551190

[28] Justyna Rój Maciej Jankowiak. Socioeconomic Determinants of Health and Their Unequal Distribution in Poland[J]. International Journal of Environmental Research and Public Health. 2021,18(20):10856. 34682597 10.3390/ijerph182010856 PMC8536126

[29] Wu Jingxian, Yang Yongmei. Inequality trends in the demographic and geographic distribution of health care professionals in China: Data from 2002 to 2016[J]. The International Journal of Health Planning and Management. 2019,34(1):e487–e508. 30238482 10.1002/hpm.2664

[30] Xu Xinglong, Zhou Lulin, Henry Asante Antwi Xi Chen. Evaluation of health resource utilization efficiency in community health centers of Jiangsu Province, China[J]. Human Resources for Health. 2018,16(1):13. 29463253 10.1186/s12960-018-0275-y PMC5819700

[31] Johan P Mackenbach Matthias Bopp, Deboosere Patrick, Kovacs Katalin, Leinsalu Mall, Martikainen Pekka, et al. Determinants of the magnitude of socioeconomic inequalities in mortality: A study of 17 European countries[J]. Health & Place. 2017,47:44–53. 28738213 10.1016/j.healthplace.2017.07.005

[32] Zhou Yong, Zhao Kaixu, Han Junling, Zhao Sidong, Cao Jingyuan. Geographical Pattern Evolution of Health Resources in China: Spatio-Temporal Dynamics and Spatial Mismatch[J]. Tropical Medicine and Infectious Disease. 2022,7(10):292. 36288033 10.3390/tropicalmed7100292 PMC9609797

